# 肿瘤细胞代谢与肿瘤转移相互关系的研究进展

**DOI:** 10.3779/j.issn.1009-3419.2014.11.07

**Published:** 2014-11-20

**Authors:** 亚龙 张, 念珍 房, 嘉琮 尤, 清华 周

**Affiliations:** 300052 天津，天津医科大学总医院，天津市肺癌研究所，天津市肺癌转移与肿瘤微环境重点实验 Tianjin Key Laboratory of Lung Cancer Metastasis and Tumor Microenvironment, Tianjin Lung Cancer Institute, Tianjin Medical University General Hospital, Tianjin 300052, China

**Keywords:** 肿瘤, 肿瘤细胞代谢, 肿瘤转移, Tumor, Metabolism of tumor cells, Tumor metastasis

## Abstract

肿瘤细胞长期处于应激的微环境中，细胞内营养物质及能量的流通速率往往高于正常细胞，肿瘤细胞代谢以此为肿瘤转移提供生物合成原料和能量。在转移性肿瘤中也发现糖代谢异常活跃、脂肪酸过度累积等异于正常细胞的代谢反应。已有研究证实对肿瘤细胞代谢进行调节可影响肿瘤转移，部分成果已应用于临床治疗，靶向肿瘤细胞代谢是抗肿瘤转移的一个积极有效的方向。本文对目前肿瘤细胞代谢与肿瘤转移相互关系和调节机制的研究进展进行综述。

## Abstract (en)

Intracellular nutrients and the rate of energy flowing in tumor cells are often higher than that in normal cells due to the prolonged stress of tumor-specific microenvironment. In this context, the metabolism of tumor cells provides the fuel of bio-synthesis and energy required for tumor metastasis. Consistent with this, the abnormal metabolism such as extremely active glucose metabolism and excessive accumulating of fatty acid is also discovered in metastatic tumors. Previous Studies have confirmed that the regulation of tumor metabolism can affect the tumor metastasis, and some of these have been successfully applied in clinical effective, positive way. Thus, targeting metabolism of tumor cells might be an effectively positive way to prevent the metastasis of tumor. So, our review is focused on the research development of the relationship between tumor metabolism and metastasis as well as the underlying mechanism.

肿瘤细胞代谢是指正常细胞在致癌因子的作用下，转变成连续进行分裂增殖的肿瘤细胞后，发生的如葡萄糖摄取量增加、乳酸堆积或者是核酸合成增加等代谢变化。肿瘤细胞三个显著的基本特征：不死性、迁移性和失去接触抑制，这些特征均与肿瘤细胞代谢有着最直接的关系。肿瘤细胞代谢比正常细胞旺盛，是为满足自身增殖和迁移需要的一个自适应的过程，细胞代谢在肿瘤细胞的发生发展以及向转移性疾病转化的过程中发挥重要作用。肿瘤细胞代谢过程中，肿瘤组织的DNA和RNA聚合酶活性均高于正常组织，核酸分解程度明显降低，DNA和RNA的含量均显著增高。以葡萄糖酵解为唯一的能量获取方式是肿瘤细胞的能量代谢特点，也是恶性肿瘤细胞一个重要的代谢特征^[1, 2]^。有研究^[3]^表明，脂代谢紊乱与肿瘤的发生与转移也具有一定的关系。

肿瘤转移是肿瘤细胞所特有属性，它是指肿瘤细胞脱离了原发病灶，通过直接蔓延、淋巴转移、血行转移和种植等转移方式，到达继发组织或者器官之后继续增殖生长，形成继发肿瘤的过程。这也造成临床上的恶性肿瘤久治不愈，手术无法完全清除肿瘤病灶的一个重要原因。针对肿瘤转移相关的分子生物学研究发现，基因突变、缺失和移位等会对细胞内的各种信号通路产生影响，主要的致癌信号通路最后都汇聚到肿瘤细胞代谢，代谢变化在肿瘤细胞的发生及发展中必不可少^[4]^。而肿瘤细胞代谢方式改变，既可以为细胞的扩增提供物质原料，还可以为细胞提供持续增值的信号^[5]^，以满足肿瘤细胞在特定的微环境中的生存需要。所以探讨肿瘤细胞代谢与肿瘤转移之间的关系可以为增加肿瘤治愈率，降低肿瘤复发风险提供新的治疗思路。我们将从肿瘤转移相关的细胞代谢改变进行综述。

## 1 糖类代谢与肿瘤转移

与正常细胞相比，肿瘤细胞摄取葡萄糖的能力更强，但他们利用这些物质的能力不如正常细胞，即使在氧含量较充足的条件下，肿瘤细胞也是主要以糖酵解的方式获得能量，同时产生大量乳酸和少量ATP，这种现象既是著名的瓦博格效应（Warburg effect）^[6]^。结果尽管糖酵解比线粒体氧化磷酸化产能效率低，但肿瘤细胞在缺氧的状态下仍可以通过糖酵解补充ATP。

### 1.1 线粒体功能的改变

线粒体是真核生物中的主要供能细胞器，同时传递调节细胞正常凋亡信号。所以，线粒体和其在细胞内的功能决定了线粒体与许多疾病，包括癌症等关系密切。从1920年开始，许多研究发现线粒体与许多具有转移性癌症发生的葡萄糖代谢的变化和失去自主凋亡能力显著相关^[7]^。在转移性癌细胞中线粒体的结构和功能发生改变，如线粒体膨胀^[8]^、葡萄糖摄入量增加、乳酸累积等，在转移性癌细胞内还发生线粒体内活性氧的生成量减少等现象。最近的一项基于瓦博格效应的研究^[9]^表明，线粒体减少葡萄糖的氧化可以增强肿瘤的转移。肿瘤细胞内除了线粒体内固有的凋亡途径外，还存在许多抗凋亡信号。在病理状态发生DNA损伤、氧化反应受到抑制、原癌基因的激活和线粒体外膜透化作用增强等变化。线粒体外膜的通透性增强可以释放一些诱导细胞凋亡的凋亡蛋白，有些凋亡蛋白，如Bax和Bid就可以增强线粒体外膜的通透性。另一方面，Bcl-2和Bcl-xL通过与凋亡蛋白结合抑制凋亡蛋白的功能。Mcl-1是Bcl-2家族的一种抗凋亡蛋白，有研究^[10]^发现它在肿瘤转移的过程中表达上升，而抗凋亡能力是肿瘤细胞转移过程中的必备条件。针对线粒体功能改变的抗肿瘤转移研究，维持线粒体正常的信号传递，增加线粒体活性氧的生成量，或调节与细胞凋亡有关的蛋白质表达等，都是在上述理论的基础上，可以进行的线粒体方面临床治疗方法的探索。

### 1.2 糖酵解途径影响肿瘤转移

糖代谢可以通过线粒体氧化磷酸化和糖酵解两种方式向细胞提供ATP，但在肿瘤细胞的线粒体氧化磷酸化供应ATP不足时，将转而通过糖酵解途径产生能量满足其生长需要。用一种永生并且非肿瘤细胞MCF10和它的三种衍生细胞亚系分别代表癌症的不同时期，与非转化MCF10细胞系相比，经过转化后的细胞系中糖酵解进行代谢的糖类百分比是降低的，亲本MCF10细胞仅通过氧化磷酸化途径分解很少一部分的葡萄糖，这完全与具有转移性的肿瘤细胞相反。在转化细胞系中，经过三羧酸循环（tricarboxylic acid cycle, TCA）循环分解的葡萄糖的量较多。这种代谢变化在具有转移性的肿瘤细胞中稳定存在，也同时是肿瘤细胞自适应表现^[11]^。口腔鳞状细胞癌患者检测发现，与病灶总糖酵解量（total lesion glycolysis, TLG）水平较低的患者相比，较高的病灶总糖酵解量与肿瘤转移的关系更为密切^[12]^。在具有转移性的肾细胞癌中，糖酵解反应是增强的，并且乳酸的产量也比正常细胞多^[13]^。

基于瓦博格效应的结果就是肿瘤细胞内的葡萄糖量增高、糖酵解活性提高和乳酸堆积^[14]^。酸性环境对肿瘤细胞具有保护作用，肿瘤细胞内糖酵解反应的增强，一个直接结果就是肿瘤细胞内乳酸含量增加，而肿瘤细胞中乳酸的水平与肿瘤的转移有直接关系^[15]^，它可以诱导与糖酵解有关的酶的表达和激活，如己糖激酶和6-磷酸果糖激酶-1（6-phosphofructokinase1, PFK1），增强肿瘤细胞中ATP的供给。肿瘤细胞可以把乳酸作为一种能量来源进行新陈代谢，还可以从周围细胞中得到乳酸来维持自身的酸性环境，以此来抗细胞凋亡和维持自身转移性^[16]^。内皮细胞的葡萄糖代谢产生乳酸，可以促进肿瘤血管形成，为肿瘤转移提供适宜环境。而部分外源性乳酸还可以使许多具有转移性的癌细胞形成浓度依赖性^[17]^。小鼠实验证实了如果抑制具有高转移性细胞的糖酵解途径，细胞的转移性明显降低^[18]^。有研究表明肿瘤细胞内集聚的乳酸是通过转化生长因子β2（transforming growth factor beta2, TGF-β2）信号通路影响肿瘤细胞转移^[17]^。所以，改变肿瘤细胞所处的酸性环境，消除肿瘤细胞的微环境优势，同时还可以减少肿瘤细胞代谢的能量来源，将是阻止肿瘤细胞转移的极为有效的手段。

### 1.3 低氧诱导因子影响肿瘤转移

近年来基于肿瘤发生和转移相关基因被大量发现，其中部分基因则是通过调节与糖类代谢有关的酶类发挥作用。早期研究^[19]^发现肿瘤细胞所处一个氧含量较正常细胞低的微环境，缺氧的微环境可以增强肿瘤细胞化疗抗性，降低患者的生存率。而低氧诱导因子1α（hypoxia-inducible factor, HIF-1α）能够在低氧的状态下被激活，引起与肿瘤代谢和转移相关的基因活化^[20]^。在自主性的缺氧和活性氧依赖性的条件下，肺癌细胞能够从肺组织向其他组织转移，在转移过程中癌细胞能瞬间上调HIF-1的表达。表明癌细胞在缺氧的微环境下，可以通过激活HIF-1因子使细胞获得向远处转移的能力^[21, 22]^。

Song等^[23]^在胃癌研究中表明，HIF-1α在缺氧环境下可以增加丙酮酸激酶-2、磷酸激酶-1、葡萄糖转运蛋白-1（Glucose transporter 1, GLUT1)和乳酸脱氢酶A（lactate dehydmgenaLse A, *LDHA*）基因的表达，促进肿瘤细胞糖类的代谢，促进细胞的生长与转移。有研究^[24]^发现，瞬间激活HIF-1可以诱导LDHA表达和E1α亚基丙酮酸脱氢酶发生磷酸化作用，说明细胞内的葡萄糖代谢途径发生变化，线粒体的氧化磷酸化转变为无氧糖酵解和乳酸发酵。当用HIF-1的抑制剂3（5’-羟甲基-2’-呋喃）-1-苄基吲唑（YC-1）抑制这一转变时，瘤内化疗时的活性氧水平增加，肺转移性肿瘤的形成也受到抑制。在158例子宫颈案例中，包括正常、宫颈上皮瘤变以及具有浸润性宫颈癌的样本。用钴的氯化物在Hela细胞和人子宫颈鳞癌细胞内制造一个低氧环境。细胞所处的正常环境和低氧环境都受到比色分析试剂盒调控，检测HIF-1α和GLUT1表达情况。结果在宫颈上皮瘤样病变组织中HIF-1α和GLUT1的表达量较正常组织高，宫颈癌内的表达量最高。分析结果发现HIF-1α与GLUT1的表达改变显著相关，与正常细胞相比，Hela细胞和人子宫颈鳞癌细胞糖酵解过程被促进。所以HIF-1α一个可能的作用机制就是增强肿瘤细胞糖酵解能力^[25]^。通过对HIF-1α的调节，降低其在肿瘤细胞中的表达，弱化HIF-α对肿瘤细胞糖酵解的增强作用，从而减少肿瘤细胞的能量摄入，降低肿瘤细胞转移能力。

## 2 脂类代谢与肿瘤转移

脂类包括脂肪、类脂及其衍生物，包括磷脂类、脂肪酸、胆固醇、甘油三酯、胆固醇酯、鞘脂和组成细胞生物膜的所必须的部分脂类，是机体最重要的供能和储能物质。有研究^[26]^证实，如果把卵巢癌细胞和脂肪细胞置于同一培养基中培养，细胞的脂类可以定向的从脂肪细胞传递到卵巢癌细胞，为细胞的扩增提供能量。脂肪的从头合成增加在肿瘤中普遍存在，是脂代谢的主要特点，常发生在肿瘤形成的早期^[27]^。有研究者^[28]^对血清中的磷脂成分进行分析，结果发现亚油酸不只是单独作用增加乳腺癌的发病风险，而且其重复去饱和作用降低了乳腺癌发病风险。将四周龄大小的雌性BALB/c小鼠分成高脂饮食组和正常饮食对照组饲养16周，然后往两组老鼠体内注入乳腺癌细胞后再按各自的饮食继续饲养。最后检测发现，正常饮食组的小鼠肺部和肝脏的肿瘤节结无论在重量还是体积上都不及高脂饮食组，同时小鼠的体重增加极少，说明高脂肪饮食有助于癌症细胞的转移^[29]^。这些研究均表明脂类代谢与肿瘤的发生与转移有密切关系。

### 2.1 胆固醇和脂肪细胞影响肿瘤转移

有研究^[30]^表明，在患病初期并且未经过任何治疗，空腹时机体内脂类的状况可用于判断病人的肿瘤是否具有转移性。肿瘤细胞在不断扩增和转移过程中对胆固醇需求不断增加，所以细胞内胆固醇代谢途径也受到调节^[31]^。胆固醇和鞘脂类聚合在细胞生物膜的微小区域内，这一结构称为脂阀。脂阀可以参与细胞内的信号传导以及介导蛋白和蛋白之间、蛋白和脂类之间的相互作用。越来越多的研究显示，脂阀与细胞表面的整合蛋白内化和循环有关，而整合蛋白在细胞转移的过程中促进细胞的粘连^[32, 33]^，胆固醇合成量的增加可能促进肿瘤细胞转移。脂阀还可以通过调节人类恶性黑色素瘤A375细胞的形态对细胞转移进行调节^[34]^。

在肿瘤细胞内，脂肪的氧化分解也会发生变化。有研究^[35]^发现，如果把乳腺癌细胞分成两组，一组与成熟的脂肪细胞一起培养，另外一组单独培养。将癌细胞从小鼠的尾静脉注入小鼠体内，之后观察发现与成熟的脂肪细胞一起培养的乳腺癌细胞在小鼠的体内转移能力明显增强。这可能是因为癌症相关的脂肪细胞可以通过脂肪分解产生脂肪酸，再将分解产生的脂肪酸传递给肿瘤细胞作为能量来源，同时再分泌脂肪因子促进肿瘤细胞的粘附、转移^[36]^。如果利用脂肪酸合成酶（fatty acid synthetase, FASN）抑制剂抑制脂肪酸合成，则肿瘤的转移也能够明显的受到抑制^[37, 38]^。所以在一个含有丰富的脂肪酸或乳酸的环境下，肿瘤细胞均能利用二者来为自身提供侵袭和转移所需要的能量，同时脂肪酸还参与肿瘤细胞转移过程。

### 2.2 脂代谢作为介质影响肿瘤转移

固醇调节元件结合蛋白（sterol regulatory element-binding proteins, SREBPs）是细胞内维持脂类代谢稳定的重要因子，并且能在恶性胶质瘤中通过调节脂类代谢抑制肿瘤生长和转移^[39]^。SREBP-1可以通过调节与脂肪酸从头合成途径有关的基因调节脂类在肿瘤细胞内的代谢，如乙酰辅酶A羧化酶（Acetyl-CoA carboxylase, *ACC*）基因和脂肪酸合成酶（fatty acid synthase, *FAS*）基因等。近期研究^[40]^表明，SREBP-1受到抑制会降低硬脂酰CoA脱氢酶-1的活性而减少不饱和脂肪酸的合成，而硬脂酰CoA脱氢酶-1是催化饱和脂肪酸向不饱和脂肪酸转化的关键酶，这造成细胞内饱和脂肪酸与不饱和脂肪酸的比例增加，不饱和脂肪酸会改变肿瘤细胞内脂类的成分，导致肿瘤细胞恶性增殖和转移。也有研究^[41]^发现，在低氧环境可以使*HIF*-*1*和*SIAH*-*2*（seven in absentia homologues）基因激活，调节谷氨酰胺代谢促进脂类的合成，从而保证肿瘤细胞的扩增需要。*p53*是发现较早的肿瘤抑制基因之一，在浆液型卵巢癌细胞中，突变后的*p53*基因可以增强脂类代谢促进肿瘤细胞转移，同时伴有诱导上皮细胞向间质细胞转化、促进脂肪细胞中的脂肪酸吸收等肿瘤细胞侵袭性变化，最终导致患者生存率下降^[42]^。p53蛋白是迄今为止研究最多的一种抑癌蛋白，而且p53蛋白的功能还在不断被揭示。*p53*突变通过促进脂类代谢而发挥作用，基于这一理论的针对*p53*突变的个性化治疗可能会是一个研究热点。

## 3 核酸代谢与肿瘤转移

核酸是生命活动最基本物质之一，在生长、遗传、变异等生命活动过程中起着决定性作用。不同生物体的不同细胞内核酸代谢具有各自的特点，在人体内肿瘤细胞和正常细胞的代谢也存在显著不同，而且在不同发展程度的肿瘤细胞之间也具有很大的区别。早期研究发现，胸苷酸和胸苷酸激酶是嘧啶核苷酸从头合成和补救合成途径的关建酶，这些酶在肿瘤细胞的激活程度高于较正常细胞，在肿瘤转移过程中这一现象更加明显^[43]^。同时分子成像技术的发展，不但可以观察细胞形态的变化，而且还可以对肿瘤细胞转移过程中核酸合成进行成像检测^[44]^。

但通过对核酸代谢和肿瘤转移等关键词检索发现，近年来针对核酸代谢的研究进行较少，尤其是与肿瘤细胞转移相关文献更是鲜有发表，笔者认为此现象可能缘于以下因素。一是因为肿瘤细胞转移过程中细胞快速增殖，包含遗传信息的传递、转录和翻译等过程，必然会引起DNA和RNA的合成增加。食物中含有足够量的核酸类物质，但他们的分解产物仅有少部分可以用于核酸的再合成。合成核酸的原料很大一部分来自糖类代谢，如核糖是来源与磷酸戊糖通路中的6-磷酸葡萄糖（G-6-P）经降解生成的5-磷酸核糖（R-5-P），5-磷酸核糖在专一的磷酸核糖焦磷酸激酶的催化下，与ATP作用生成5-磷酸核糖焦磷酸(Phosphoribosyl pyrophosphate, PRPP)用于单核苷酸的合成。糖类代谢是肿瘤转移过程中核酸合成的物质基础，这可能是造成针对核酸代谢研究较少的一个原因。其次，因为目前分子生物学的发展，寻求的是造成某种结果的根本原因。针对肿瘤转移的研究，焦点大多在基因层面，如微小RNA（microRNAs, miRNA）对肿瘤转移的调节，长链非编码RNA（long noncoding RNA, lncRNA）与肿瘤转移的相关性等。而核酸的合成和分解可能是这些调节过程的结果，而在代谢的大分子层面少有研究。但是，若在核酸代谢水平研究肿瘤转移，抑制或者激活某些与核酸合成相关的酶的活性，如磷酸核糖酰胺转移酶、5-磷酸核糖焦磷酸激酶等，可能会成为肿瘤治疗的一个新靶点。

## 4 酶与肿瘤转移

在转移性的肿瘤细胞中，酶的催化作用在核酸、糖类、脂类的代谢中更为重要。例如葡萄糖-6-磷酸脱氢酶是磷酸戊糖途径的调节酶，不断的合成核苷酸和脂类，满足肿瘤转移的物质和能量需要，所以葡萄糖-6-磷酸脱氢酶也成为抗肿瘤的一个潜在靶点^[45]^。氧糖基化蛋白可以增强糖基转移酶活性，因此也是卵巢癌细胞发生细胞迁移的主要标志。因为乙酰氨基半乳糖转移酶14（polypeptide N-acetylgalactosaminyltransferase 14, GALNT14）糖基化与跨膜粘蛋白-13有关，他们在卵巢癌细胞中含量较高。通过小干扰RNA（small interfering RNA, siRNA）敲除*GALNT14*基因，可抑制细胞转移。表明GALNT14可以通过影响跨膜粘蛋白-13影响肿瘤转移^[46]^。已有研究发现乙酰肝素酶在肿瘤的侵袭和转移过程中发挥重要作用。若用乙酰肝素酶抑制剂OGT2115和低分子肝素抑制乙酰肝素酶的表达，肿瘤细胞的侵袭和转移也会受到抑制^[47]^。还原型烟酰胺腺嘌呤二核苷酸磷酸（reduced form of nicotinamide-adenine dinucleotide phosphate, NADPH）氧化酶是活性氧类（reactive oxygen specie, ROS）的主要来源，而体内产生的ROS可以诱发内皮细胞的凋亡，这正是肿瘤细胞脱离循环系统，继而转移到其他组织和器官的前提条件^[48]^。血管紧张素转化酶-2是肾-血管紧张肽系统的关键酶，它可以降低肺癌细胞的转移^[49]^。腺苷酸活化蛋白激酶（adenosine monophosphate-activated protein kinase, AMPK）可以调控细胞转移，但是在癌症细胞中的功能还不明确。在卵巢癌异种移植模型中敲除AMPK基因可以显著抑制肿瘤向肺部转移，通过溶血磷脂酸（lysophosphatidic acid, LPA）诱导的AMPK激活也可以诱发卵巢癌细胞转移^[50]^。AMPK-α2可有效缓解肿瘤转移造成的肝脏损伤，包括缓解肝脏的缺糖损伤和线粒体介导的ROS产物生成抑制^[51]^。上述表明不同酶在肿瘤转移过程中通过不同途径发挥重要作用，通过对酶在肿瘤细胞转移过程中调节作用的研究，可以特异性降低核苷酸和脂类的合成速率，降低肿瘤细胞的能量利用率。利用酶抑制剂抑制转移相关酶的活性，这也是临床最常用治疗方法之一。

## 5 问题与展望

细胞代谢是一切生命体的基础，肿瘤转移更是建立在肿瘤细胞为适应细胞生长和转移需要而进行自身代谢改变的基础上。肿瘤细胞代谢在肿瘤转移过程中会进行自我调整，但是肿瘤细胞代谢会形成哪些新的途径？例如，减少与肿瘤生长和转移有关的氨基酸的产生，同时可以增加促进机体蛋白质合成或能够刺激机体免疫机制的氨基酸的合成，从而达到抑制肿瘤转移的目的。或降低与糖酵解有关酶的活性，或者控制食物中能量的供给，使肿瘤细胞长期处于一个能量匮乏的微环境中。但是就目前对肿瘤细胞代谢的研究，从代谢的角度治疗转移性肿瘤还存在许多不明确因素。

首先，肿瘤转移复发一直是治疗肿瘤的一大难题。舒尼替尼和索拉菲尼可以通过阻止血管的生成达到抑制肿瘤生长的效果，但是当药物停用以后，肿瘤又获得重新生长，血管再生功能修复，同时乳酸再次积聚增加，脂类合成能力增强。以上代谢变化最终导致肿瘤转移性传播急剧增加。如果当停药后，继续辅以降低脂肪酸合成的治疗措施，则可以抑制肿瘤的再次生长^[52]^。因此肿瘤细胞转移能否可以直接通过对代谢水平各种物质的控制实现转移抑制作用？如果通过调节肿瘤细胞代谢，肿瘤转移被抑制后是否会再次发生？其次，目前在抑制肿瘤转移方面，适用基因调控，或者选择代谢水平控制糖类或其他物质的分解合成并无明确判断，适用性还有待进一步研究。有研究人员利用靶向肿瘤代谢的纳米脂质体，有效的减缓甚至阻止肿瘤细胞的转移^[53]^，所以也再次说明在肿瘤细胞代谢方面还将有许多未知的靶点可以抑制肿瘤细胞转移。

综上所述，尽管肿瘤转移是一个复杂的过程，代谢改变与肿瘤转移之间的关系已经明确，类似于高胆固醇的环境有利于肿瘤转移等代谢改变已经被证实，但是其影响肿瘤细胞转移的机制仍不清晰。也有人提出，线粒体的氧化代谢将成为抑制肿瘤转移的一个潜在靶点，对肿瘤转移治疗具有更大的潜在价值^[54]^，针对肿瘤细胞线粒体的研究可能会成为一个重要方向，这些问题都需要我们继续进行深入的研究发现。并且与尝试在基因水平阻止肿瘤转移相比，基因功能及相互之间的作用需要投入大量资源，而调节肿瘤细胞代谢过程防止肿瘤转移将更加直接和高效。随着对肿瘤细胞代谢研究的深入，靶向代谢水平的抗肿瘤转移治疗也会为临床提供新的诊断和治疗方法。
